# Q methodology with cognitive interviewing to rank the importance of informational items in a patient information leaflet

**DOI:** 10.1186/1745-6215-16-S2-P76

**Published:** 2015-11-16

**Authors:** Karen Innes, Katie Gillies, Seonaidh Cotton, Marion Campbell

**Affiliations:** 1 University of Aberdeen, Aberdeen, UK

## 

Patient Information Leaflets (PILs) are required for UK studies. Health Research Authority (HRA) guidance lists 36 topic areas for inclusion in PILs. However, there is limited evidence about whether stakeholders believe these items to be of importance when considering participation in a Randomised Controlled Trial (RCT). This study will identify and assess which items of information trial stakeholders rank as most important and reasons for this.

Our study uses aspects of Q-methodology (a card sort technique) and simultaneous cognitive interviews (think aloud). This mixed methods approach captures data on subjective opinions held around a particular area of interest. The card sort technique provides participants with a set of ‘cards’ (statements describing specific information items) which they rank according to their opinion of relative importance on a specially formatted grid which captures the relative rankings. While the participant completes the card sort, they are encouraged to use the think-aloud technique to verbalise their thoughts.

In this study, the statements included on the cards relate to the information items recommended by HRA for inclusion in a PIL. Twenty trial stakeholders (10 potential trial participants and 10 research nurses) will complete the card sort within one-to-one think-aloud interviews. To contextualise the card sort, participants are asked to imagine they have been approached to participate in a phase III RCT comparing treatments A and B for a chronic condition. The card set and study methodology have been piloted. We will present an overview of the methods described above and preliminary findings from our study.

